# Gender difference in health related quality of life and associated factors among people living with HIV/AIDS attending anti-retroviral therapy at public health facilities, western Ethiopia: comparative cross sectional study

**DOI:** 10.1186/s12889-018-5474-x

**Published:** 2018-04-23

**Authors:** Delelegn Yilma Gebremichael, Kokeb Tesfamariam Hadush, Ermiyas Mulu Kebede, Robel Tezera Zegeye

**Affiliations:** College of medicine and health sciences, Department of Public Health, Ambo University, P.O. Box 19, Ambo, Ethiopia

**Keywords:** HIV/AIDS, Health related quality of life, Antiretroviral therapy

## Abstract

**Background:**

Though HIV/AIDS has multidimensional consequences on quality of life, there is a gap in measuring and monitoring health related quality of life of HIV/AIDS patients. Hence, this study intended to measure health related quality of life domains and associated determinants among people living with HIV/AIDS in western Ethiopia.

**Methods:**

A comparative cross-sectional study was conducted among 520 HIV/AIDS patients on anti-retroviral therapy in public health facilities in West Shoa Zone, Western Ethiopia from April to May, 2016. Participants were selected using simple random sampling method. Quality of life was measured using WHOQOL-HIV BREF and depression was assessed using Beck Depression Inventory, Second Edition (BDI-II). Data were analyzed using SPSS version 22. An independent sample t-test was used to compare quality of life domains between men and women and logistic regression analysis was used to determine independent predictors.

**Results:**

Females had significantly lower quality of life in physical, psychological, independence and environmental domains as compared with males except social relationship and spiritual domains. Depressed HIV patients had significantly lower quality of life in all domains as compared with HIV infected patients without depression in both genders. Malnutrition and anemia were significantly associated with poor physical, psychological, independence and environmental domains. Anemic women had 1.9 times lower independence quality of life compared with women who had no anemia (AOR = 1.9, 95%CI: 1.4, 3.5). Tuberculosis was also predictor of physical, psychological, independence and social domains in both genders. TB/HIV co-infected females had 2.0 times poorer environmental health compared to only HIV infected females (AOR = 2.0, 95%CI: 1.2, 3.5). Family support, education and occupation were also independent significant predictors of QOL domains in both genders. In females, residence was significantly associated with independence (AOR = 1.8, 95%CI: 1.2–3.8) and environmental (AOR = 1.5, 95%CI: 1.1–3.2) domains.

**Conclusions:**

Females had significantly lower quality of life compared with males. The findings indicted poor socio-economic status and co-infections significantly associated with poor quality of life among HIV/AIDS patients. So, due emphasis should be given to improve socio-economic status and enhance integrated early detection and management of malnutrition, depression, tuberculosis and anemia among HIV/AIDS patients in Ethiopia.

## Background

HIV/AIDS remains one of the most common public health problem in developing countries like Ethiopia [1]. According to global burden of diseases report, HIV/AIDS accounted for 1.1 million deaths in the world in 2016. it was the greatest single cause of years of life lost (YLL), contributed to 59.8 million years of life lost [2]. HIV/AIDS has been a major cause of morbidity and mortality in resource-poor settings, especially in sub-Saharan Africa where around 25 million people were living with HIV [1]. Ethiopia is among the countries most heavily affected by HIV/AIDS which poses threat to the country’s overall development. In 2016, an estimated 740, 251 people were living with the virus and 442,895(59.8%) of them were females [3]. Moreover, AIDS accounted for an estimated 34% of young adult deaths and 632,670 orphanages in the country [4]. Specifically, In the study area (West Shewa zone) 18,642 people were living with HIV/AIDs(PLWHA) in 2016.

HIV/AIDS has changed individual’s lifestyles and quality of life. It leads to physiological, physical, psychological, and sociocultural problems that are caused by many factors such as symptoms of the virus, side effects of ART, and opportunistic infections [5, 6]. Studies have shown that HIV/AIDS has multidimensional consequences: personal suffering such as discomfort associated with the disease’s progression, the social impact of the diagnosis, the emotional consequences of dealing with the diagnosis, and related stigma [7–9]. Studies have identified disease-related factors such as CD4 count, viral burden, HIV disease stage, infections [6, 9, 10] and psychosocial factors like social support, coping, and disclosure [7] as predictors of quality of life among people living with HIV/AIDS. Depression has also been associated with changes in general health perceptions, emotional well-being and QOL domains in HIV patients [11, 12]. Additionally, several socio-demographic characteristics such as age, gender, education and employment were also factors associated with lower QOL [6, 8, 13]. Hence, finding ways of mitigating these factors and consequences makes quality of life(QOL) in PLWHA a relevant issue of health care [14].

The information on gender-specific differences in QOL outcomes has been controversial. On the whole, several studies have shown that females had significantly lower quality of life compared with males [6, 15–18]. On the other hand, in studies conducted in western countries gender showed no major impact on QOL [19, 20]. However, in settings with limited health care resources, heavy HIV-related stigma and discrimination like Ethiopia, women’s entrenched economic and social inequality within their relationships with men that could constrain their ability to access to care, treatment and supportive services. These barriers could make women more vulnerable to the physical and psychological burden of HIV [4, 21]. A study examined gender differences in quality of life in Ethiopia reported lower quality of life among women compared to men [22] Other studies conducted on QOL in patients with HIV /AIDS have reported overall low QOL scores [5, 23–26]. However, more specific conclusions about gender differences difficult to be made from these studies due to their limitations that included: lack of comparison group and underpowered since single proportion was used to determine sample size which made the samples difficult to be representative and only over all QOL was reported (they did not report gender difference in individual QOL domains and predictors).It would be important to determine if there are gender differences in QOL domains from comparative studies in order to understand differences in how men and women are affected by HIV/A IDS, and potentially identify areas where interventions could improve patients’ quality of life.

In general, evidence showed that there appears to be very limited research measuring outcomes in terms of improved health related quality of life in Africa including Ethiopia [27]. HIV/AIDS remains one of the key challenges for the overall development of the country [4] due to high rate of morbidity and mortality as well as its negative impact on individuals well-being and quality of life [3, 22]. In addition, the presence of women’s economic dependency on men and relatively high stigma and discrimination among HIV infected women could limit their access to treatment and supportive services [21]. Moreover, investigation of contextual gender specific factors associated with QOL would help policy makers to plan gender specific comprehensive and effective HIV care service to improve the lives of PLWHA [28]. However, gender differences in QOL domains and respective predictors not well documented in Ethiopia in general and specifically in the study area. Hence, this study intended to assess sex-specific social, physical, mental, independence, spiritual and environmental domains and their determinants among HIV/AIDS patients in order to understand how people’s lives are affected by HIV infection, to help health providers in making the best choices in patient cares and aid to design palliative programs accordingly.

## Methods

### Study design and participants

The study used institution based comparative cross-sectional study design and the study population were HIV/ADIS patients aged 18 years or older who have been attended ART at public health facilities in West shoa Zone from April to May,2016, western Ethiopia. Sample size was determined using the following formula which helps to compare between two means (Equal sample sizes) male versus females with consideration of power 80%, 95% CI, a 1:  1 ratio and Overall HRQOL mean 81.2 (SD ± 14.2) for males and 77.1 (SD ± 17.4) for females [5].

$$ {n}_1={n}_2=\frac{{\left({Z}_{\alpha /2}+{Z}_{\beta}\right)}^2\left({\sigma_1}^2+{\sigma_2}^2\right)}{\varDelta^2} $$

∆ = /μ1-μ2/ = mean difference between the two respective groups

(μ1, σ1^2^) and (μ2, σ2^2^) are the means and variances of the two respective groups

$$ \mathrm{n}1=\mathrm{n}2=\frac{{\left(1.96+0.84\right)}^2\left({(14.2)}^2+{(17.4)}^2\right)=236}{{\left(81.2\hbox{--} 77.1\right)}^2} $$

After adding 10% non-response rate the final sample size was 520 (260 males and 260 females). From 24 health facilities that provide ART services in west shoa zone, Ambo Hospital and five health centers (Awaro, Ginch, Bako, Holeta and Guder) were randomly selected using lottery methods. Then the study participants were selected proportionally from each health facilities using simple random sampling method using their ART unique number.

### Data collection and measurements

HRQOL was measured using the interviewer administered World Health Organization’s Quality of Life HIV short form instrument (WHOQOL-HIV BREF) [29]. The WHOQOL-HIV BREF instrument produces six domain scores and contains 31 items. For each item there is a five-point Likert scale where 1 indicates low or negative perceptions and 5 high or positive perceptions. These items contain six domains: physical health (4 items), psychological well-being (5 items), social relationship (4 items), environmental health (8 items), level of independence (4 items), and spiritual health (4 items). There are two items that examine general quality of life: question 1 asks about an individual’s overall perception of quality of life and question 2 asks about an individual’s overall perception of his or her health. The physical health domain contained information on presence of pain, energy, and sleep. The psychological domain consisted of negative and positive feelings, self-esteem, and thinking. The social domain covered social support, personal relationship, and sexual activity. Mobility, work capacity, and activities were included in the level of independence. Financial issues, home and physical safety and security, and participation in leisure activities were included under the environment domain. The spirituality domain did contain questions about death and dying, forgiveness and blame, and concern about the future. The suggested reference time frame of QOL experienced within two weeks was used in this study [28]. Depression was assessed using BDI-II (Beck Depression Inventory, Second Edition) [30]. It consists of 21 questions scaled from 0 to 3, so the lowest possible total score is zero and the highest possible total for the whole test would be sixty-three. BDI-II: 0–13 points considered no depression and above 13points considered depression.

Anthropometrical measurements (weight, height) were taken to assess nutritional status of patients. Weight of the participants was measured in light clothing and bare foots using standard beam balance to the nearest 0.1 kg and the scale was checked at zero before each measurement. Height was measured using the standard scale. The subjects were asked to remove their shoes, stood erect, and positioned at the Frankfert plane with feet together and knees straight. The heels, buttocks, shoulder blades and the back of the head (occiput) were in touch against the vertical stand of the stadio meter and the values were recorded to the nearest 0.1 cm. Body Mass Index (BMI) (Weight (kg)/ Height^2^ (m) was computed using the weight and height measurements to assess nutritional status. The standard cut-offs were used: < 18.5 kg/m^2^ underweight, 18.5–24.9 kg/m^2^ normal, and greater than or equals to 25.0 kg/m^2^ was considered overweight. Clinical, laboratory and ART data were collected through reviewing records from ART entry registration book and individual follow-up form using pretested data collection form. Data on WHO clinical stages, CD4 count, reported side effects, drug adherence, and functional status of participants were extracted by six trained nurses from medical charts in the ART clinics. Data about socio-demographic characteristics, quality of life, depression and family support from family members in terms of psychological, financial, material or ART treatment support were collected through face to face interview using pretested interviewer administered questionnaires.

### Data analysis

Data were entered to Epi-Info version 3.5.1 statistical software for windows and analyzed using SPSS version 22 software. The WHOQOL- HIV BREF [29] was used to produce a QOL profile derived from six domain scores denoting facets of an individual’s perceived QOL. The mean score of items within each domain were used to calculate the domain score. Mean scores then multiplied by four to make domain scores comparable with the scores used in the WHOQOL-100, a commonly used scale. The instruments’ guidelines for checking and cleaning data and computing domain scores were rigorously followed. Participants characteristics were described in terms of mean (Standard deviation) and median (inter quartile range) for continuous data and frequency distribution for categorical data.

Pearson correlation coefficient(r) was used to evaluate correlation between the domains of quality of life. Independent samples t-test was used to compare domains of quality of life between men and women. Bonferroni procedure was used to declare significance difference in correlated domains of quality of life between men and women in order to minimize type one error. Overall significance level was divided by the number of two-way comparisons made (0.05/8 = 0.006) and significance level 0.006 was used declare the significance difference between the two means. The Cronbach α was calculated to determine the internal consistency of the different domains of the WHOQOL-HIV instrument. Mean was used as a cutoff point for quality of life domains because of a normal distribution of the scores. To determine independent predictors of QOL, participants were divided into two groups based on the mean score of the facet (range 1 to 5) because the WHOQOL- HIV BREF denoted facets of an individual’s perceived QOL as 1 = Very poor, 2 = Poor, 3 = Neither poor nor good, 4 = Good and 5 = Very good, mean score ≤ 3.0 indicates poor QOL and mean score of domain (range 4 to 20) since mean scores multiplied by four to make domain scores comparable with the scores used in the WHOQOL-100, a commonly used scale, domain mean score ≤ 12.0 indicates poor QOL. Therefore, Participants with mean score > 12.0 were categorized as having good QOL and their counterparts mean score ≤ 12.0 as having poor QOL. Bivariate and multivariate logistics analysis were carried out to assess independent predictors of HRQOL domains in both genders. Goodness of fitness of the final model was checked using Hosmer and Lemeshow statistic. Crude and adjusted odds ratios with their 95% Confidence Interval (CI) were estimated and *P*-Value less than 0.05 was used to declare presence of significant association between HRQOL domains and covariate.

## Results

### Socio-demographic characteristics of the study participants

A total of 251 males and 254 females responded to the study making a response rate of 97.1%. The mean age(SD) for males was 38.5 (±8.5) years while for females 33.8(±8.4) years. Majority of the study participants were rural resident; 177(70.5%) males and 158(62.2%) females. One hundred eighty-six (74.1%) males and 158(62.2%) females were married. Regarding education, 163(64.9%) males and 141(55.5%) females were literate. Two hundred thirty-one (92.0%) males and 210(77.2%) females were employed (Table 1).

**Table 1:** Sociodemographic and Clinical characteristics of people living with HIV/AIDS in public health institutions, western Ethiopia, 2017

Sociodemographic Characteristics		Male	Female	Total
		Number (%)	Number (%)	Number (%)
Mean (±SD) age		38.5 ± 8.5	33.8 ± 8.4	36.2 ± 8.8
Residence	Rural	177(70.5)	158(62.2)	335(66.3)
	Urban	74(29.5)	96(37.8)	170(33.7)
Marital status	Single	36(14.3)	51(20.1)	87(17.2)
	Married	186(74.1)	158(62.2)	344(68.1)
	Widowed	20(8.0)	31(12.2)	51(10.1)
	Divorced	9(3.6)	14(4.5)	23(4.6)
Ethnicity	Oromo	221(88.0)	211(83.1)	432(85.5)
	Amhara	22(8.8)	38(15.0)	60(11.9)
	Others	8(3.2)	5(2.0)	13(2.6)
Religion	Protestant	87(34.7)	95(37.4)	182(36.0)
	Orthodox	151(60.2)	147(57.9)	298(59.0)
	Muslim	10(4.0)	9(3.5)	19(3.8)
	Others	3(0.6)	3(0.6)	6(1.2)
Education	Illiterate	88(35.1)	113(44.5)	201(39.8)
	Literate	163(64.9)	141(55.5)	304(60.2)
Occupation	Employed	231(92.0)	210(77.2)	441(87.3)
	Unemployed	20(8.0)	44(17.3)	64(12.7)
WHO stage	Stage I	31(12.4)	23(9.1)	54(10.7)
	Stage II	35(13.9)	47(18.5)	82(16.2)
	Stage III	124(49.4)	122(48.0)	246(48.7)
	Stage IV	61(24.3)	62(24.4)	123(24.4)
CD4 count	median IQR (cells/μl)	209(142–334)	186(136–286)	202(138–310)
ART Duration	median (IQR) (month)	47(27–74)	45(23.7–69.8)	46(25–72)
BMI(kg/m2)	≥18.5	202(80.5)	179(70.5)	381(75.4)
	< 18.5	49(19.5)	75(29.5)	124(24.6)
Depression	Normal	186(74.1)	159(62.6)	345(68.3)
	depressed	65(25.9)	95(37.4)	160(31.7)
Tuberculosis	Yes	25(10.0)	33(13.0)	58(11.5)
	No	226(90.0)	221(87.0)	447(88.5)
Anemia	Normal	191(76.1)	178(70.4)	369(73.2)
	Anemic	60(23.9)	75(29.6)	135(26.8)
Psychosocial support	Yes	154(61.4)	138(54.3)	292(57.8)
	NO	97(38.6)	116(45.7)	213(42.2)

Showed the sociodemographic and clinical characteristics of people living with HIV/AIDS (Study participants). A total of 251 males and 254 females responded to the study making a response rate of 97.1%. The mean age(SD) for males was 38.5 *(*±8.5) years while for females 33.8(±8.4) years. Majority of the study participants were rural resident; 177(70.5%) males and 158(62.2%) females. One hundred eighty-six (74.1%) males and 158(62.2%) females were married. Regarding education, 163(64.9%) males and 141(55.5%) females were literate. Two hundred thirty-one (92%) males and 210(77.2%) females were employed. Half of the study participants were at clinical WHO stage III, 124(49.4%) of males and 122(48.0%) of females. Median CD4 count (IQR) was 209(142–334) for males and 186(136–286) for females. Concerning nutritional status; 49(19.5%) of males and 75(29.5%) of females were undernourished. Sixty-five (25.9%) of males and 95(37.4%) of females were depressed. Twenty-five (10%) of males and 33(13%) of females had tuberculosis. Sixty (23.9%) of males and 75(29.6%) of females were Anemic

### Clinical profiles, nutritional and depression status of the study participants

Half of the study participants were at clinical WHO stage III, 124(49.4%) of males and 122(48%) of females. Median CD4 count (IQR) was 209(142–334) for males and 186(136–286) for females. Concerning nutritional status; 49(19.5%) of males and 75(29.5%) of females were undernourished. Sixty-five (25.9%) of males and 95(37.4%) of females were depressed. Twenty-five (10%) of males and 33(13%) of females had tuberculosis. Sixty (23.9%) of males and 75(29.6%) of females were Anemic. 154(61.4%) males and 138(54.3%) females received psychosocial support from family members or friends or organizations (Table 1).

### Internal consistency of the WHOQOL-HIV questionnaire and Beck depression inventory tool

The internal consistency of the *WHOQOL-HIV* tool was high (Cronbach’s α = 0.88) and to measure internal consistency, the Cronbach’s alpha was also calculated for each domain of the instrument. Most domains of the WHOQOL-HIV had a high value of Cronbach’s alpha (α > 0.7) ranging from 71 for psychological domain to 83 for environmental domain. But, medium internal consistency was observed in social relationship domain (α = 0.64) physical (α =0.75). Inter domain correlations showed that there were statistically significant associations between domains. Strong correlation was found between level of physical health and independence (*r* = 0.8, *p*-value = 0.001), and weak correlation was observed between spiritual and environmental (*r* = 0.3, p-value < 0.01). The correlation between items in the Beck Depression Inventory scale ranged from 0.52 to 0.76 and the internal consistency of the Beck Depression Inventory tool was high (Cronbach’s α = 0.92). We found strong correlation between the QOL domains and the Beck Depression Inventory scale. Strong correlation was found between the Psychological domain and the Depression Inventory scale (correlation coefficient, *r* = − 0.6) followed with social (*r* = − 0.5). Physical (*r* = − 0.5), level of independence (*r* = − 0.4), spiritual (*r* = − 0.4) and environmental (*r* = − 0.3) domains with the Beck Depression Inventory scale (*P*-value = 0.001).

### Gender difference in mean score of health related quality of life domains

The result showed that average scores of females for all the six domains and two general questions were lower than the males score on the 4–20 scales. There was significant difference in quality of life between males and females for all domains and two general Questions except the Social relationships and spiritual/personal belief domains (*p*-value < 0.006). The overall mean (± SD) quality of life perception was 3.5(±1.1) for males and 3.0(±1.0) for females. Similarly, the mean (± SD) score for general health perception score was 4.2(±1.2) for males and 3.5(±1.2) for females. Mean (SD) Physical domain was significantly different between males and females; 15.3(±3.2) for males and 13.9(±2.6) for females. Similarly, males and females were significantly different in Psychological Domain; with mean (± SD) of 14.1(±4.0) for males and 12.6(±3.0) for females. Mean Level of independence domain for females 12.1(±2.5) was also significantly lower than males 13.7(±3.3). Social relationship was the second lowest QOL domain, with mean (± SD) score of 12.0(±2.8) for males and 11.9(±2.5) for females. Spiritual/PB domain was the highest HRQOL domain with mean (± SD); 15.7(±3.2) for males and 15.5(±3.0) for females and environmental domain was the lowest with mean (± SD) score; 11.4(±3.4) for males and 10.4(±2.6) for females (Table 2).

**Table 2:** Gender difference in mean score of HRQOL domains of PLWHA on ART in public health institutions, western Ethiopia, 2017

HRQOL Domains	Mean ± SD	Mean ± SD	*t*-value	*P*-value
	Male (= 251)	Female (= 254)		
Overall HRQOL	3.5(1.1)	3.0(1.0)	4.4	0.001
General health perception	4.2(1.2)	3.5(1.2)	6.7	0.001
Physical	15.3(3.2)	13.9(2.6)	5.9	0.001
Psychological	14.1(4.0)	12.6(3.0)	6.2	0.001
Level of independence	13.7(3.3)	12.1(2.5)	5.4	0.001
Social relationships	12.0(2.8)	11.9(2.5)	1.0	0.322
Environment	11.4(3.4)	10.4(2.6)	3.9	0.001
Spiritual/personal belief	15.7(3.2)	15.5(3.0)	1.5	0.132

Displayed the gender difference in mean score of health related quality of life domains of PLHIV on ART in public health institutions, western Ethiopia. The result showed that average scores of females for all the six domains and two general questions were lower than the males score on the 4–20 scales. There was significant difference in quality of life between males and females for all domains and two general questions except the Social relationships and spiritual/personal belief domains (*p*-value < 0.006)

### Predictors of health related quality of life domains

Sex-specific multiple regression analyses revealed that education, occupation, malnutrition, depression, tuberculosis, anemia and family support were independent predictors of health related quality of life domains in both genders. In addition, rural residence was also significantly associated with QOL domains in females. Illiterate individuals reported significantly lower QOL in independence (AOR = 2.5(95%CI:1.3, 4.2), social (AOR = 1.5(95%CI:1.1, 4.4) and environmental quality of life in both female and male respondents. Similarly, employment was also significantly associated with physical, psychological, social and environmental domains in both genders. Among females, rural residents showed 1.8 and 1.5 times lower QOL in independence (AOR = 1.8(95%CI: 1.2, 3.8) and environmental (AOR = 1.5(95%CI: 1.1, 3.2) domains than urban residents respectively (Tables 3 & 4).

**Table 3:** Predictors of health related quality of life domains of male HIV patients on ART in public health institutions, western Ethiopia, 2017

Variables	Physical	Psychological	Independence	Social	Environment	Spiritual
	AOR (95% CI)	AOR (95% CI)	AOR (95% CI)	AOR (95% CI)	AOR (95% CI)	AOR (95% CI)
Education						
Literate	1	1	1	1	1	1
Illiterate	1.3(0.5–2.8)	1.1(0.6–3.1)	2.0(1.2–3.7) *	1.8(1.3–4.2) *	1.5(1.1–4.4) *	1.1(0.8–3.0)
Occupation						
Employed	1	1	1	1	1	1
Unemployed	2.2(1.4–5.1) *	2.0(1.2–4.8) *	1.6(0.7–3.5)	1.5(1.1–5.0) *	1.7(1.3–4.9) *	1.3(0.9–3.5)
BMI(kg/m2)						
< 18.5	2.5(1.7–5.4) *	1.7(0.8–4.7)	1.6(1.4–3.8) *	1.3(0.6–3.2)	2.6(1.8–6.1) *	1.1(0.3–4.2)
≥18.5	1	1	1	1	1	1
Depression						
Yes	3.1(2.0–5.9) *	3.7(2.4–7.5) *	2.8(1.9–6.4) *	3.6(2.3–6.5) *	2.4(1.6–4.7) *	1.5(1.1–2.9) *
No	1	1	1	1	1	1
Tuberculosis						
Yes	2.3(1.3–6.8) *	2.0(1.2–4.9) *	1.4(1.7–4.1) *	2.1(1.1–5.7) *	1.6(0.8–3.5)	1.1(0.5–2.8)
No	1	1	1	1	1	1
Anemia						
Yes	2.5(1.8–6.2) *	1.8(1.3–4.2) *	1.5(0.7–3.9)	1.1(0.4–2.3)	1.5(1.3–3.4) *	1.6(0.8–4.0)
NO	1	1	1	1	1	1
Family support						
Yes	1	1	1	1	1	1
NO	1.6(0.9–2.5)	5.7(4.2–10.6) *	1.6(0.8–4.2)	4.2(3.1–8.5) *	1.3(0.8–3.2)	2.0(1.5–3.6) *

Showed predictors of health related quality of life domains among men and women patients respectively. Education, occupation, undernutrition, depression, tuberculosis, anemia and family support were independent predictors of health related quality of life domains in both genders. in addition, rural residence was also significantly associated with QOL domains (*p*-value < 0.05)

**Table 4:** Predictors of health related quality of life domains of female HIV patients on ART in public health institutions, western Ethiopia, 2017

Variables	Physical	Psychological	Independence	Social	Environment	Spiritual
	AOR (95% CI)	AOR (95% CI)	AOR (95% CI)	AOR (95% CI)	AOR (95% CI)	AOR (95% CI)
Residence						
Urban		1	1	1	1	1
Rural	1.4(0.1–2.7)	1.5(0.6–4.4)	1.8(1.2–3.8) *	1.7(0.8–3.5)	1.5(1.1–3.2) *	1.3(0.6–2.9)
Education						
Literate	1	1	1	1	1	1
Illiterate	1.2(0.7–2.6)	1.2(0.8–2.5)	2.5(1.3–4.2) *	2.4(1.6–4.6) *	2.1(1.5–4.0) *	1.5(0.8–3.3)
Occupation						
Employed	1	1	1	1	1	1
Unemployed	2.8(1.6–5.3) *	2.2(1.3–3.7) *	1.7(0.9–3.2)	1.6(1.1–3.4) *	2.1(1.3–4.7) *	1.5(0.4–2.8)
BMI(kg/m2)						
< 18.5	3.5(1.9–5.2) *	2.2(1.3–4.3) *	2.0(1.3–3.7) *	1.5(0.7–3.2)	3.8(2.2–7.4) *	1.4(0.5–2.6)
≥ 18.5	1	1	1	1	1	1
Depression						
Yes	3.8(2.4–5.7) *	4.5(2.7–7.9) *	3.7(2.3–5.6) *	4.0(2.5–6.9) *	2.9(1.6–4.5) *	1.9(1.1–3.5) *
No	1	1	1	1	1	1
Tuberculosis						
Yes	2.7(1.3–7.8) *	2.5(1.2–6.7) *	1.8(1.3–4.4) *	2.6(1.4–4.8) *	2.0(1.2–3.5) *	1.2(0.6–3.3)
No	1	1	1	1	1	1
Anemia						
Yes	3.2(1.7–6.2) *	2.8(1.5–3.7) *	1.9(1.4–3.5) *	1.1(0.7–2.2)	1.8(1.5–4.2) *	1.5(0.6–3.0)
NO	1	1	1	1	1	1
Family support						
Yes	1	1	1	1	1	1
NO	1.8(1.2–3.3) *	7.8(4.7–12.6) *	2.1(0.9–3.2)	5.5(3.4–8.0) *	1.5(0.9–3.6)	2.3(1.6–3.9) *

NB. * = *P*-Value < 0.05

Showed predictors of health related quality of life domains among men and women patients respectively. Education, occupation, undernutrition, depression, tuberculosis, anemia and family support were independent predictors of health related quality of life domains in both genders. in addition, rural residence was also significantly associated with QOL domains (*p*-value < 0.05)

Physical, independence and environmental QOL domains were significantly reduced among undernourished patients in both genders. Furthermore, undernourished females had 2.2 times poorer psychological QOL compared with well-nourished counter parts (AOR = 2.2(95%CI: 1.3, 4.3). Similarly, statistically significant differences were found in physical, psychological and environmental QOL domains with anemia status in both male and female patients. In addition, anemic women had 1.9 times lower independence QOL compared with women who had no anemia (AOR = 1.9(95%CI: 1.4, 3.5). For both genders, tuberculosis was the common predictor of physical, psychological, independence and social QOL domains. Besides, TB/HIV co-infected females had 2.0 times poorer environmental QOL as compared to only HIV infected females (AOR = 2.0(95%CI: 1.2, 3.5). Depressed PLWHA had significantly lower QOL in all domains as compared with HIV infected patients without depression in both genders. Likewise, Family support was also a common predictor for psychological, social and spiritual QOL domains in both male and female patients. Moreover, women who received family support were found to have 1.8 times higher physical QOL compared with women who lacked family support (AOR = 1.8(95%CI: 1.2, 3.3). However, age, marital status, CD4 count and WHO clinical stage were not significantly associated with quality of life (Tables 3 & 4).

## Discussion

This study revealed that females had significantly lower quality of life in physical, psychological, independence and environmental domains as compared with males. This finding was consistent with study conducted in Ethiopia reported that females had significantly lower QOL for all domains except the social relationship domain [22]. The study done in Vietnam also found that females had significantly lower scores than males in environmental and psychological domains [15]. Cross sectional studies conducted in Estonia and Nepal also supported this finding that female had lower QOL in physical, psychological, social and environmental health [16, 17]. The possible reason for females scoring lower QOL than males could be that many women living with HIV are burdened by responsibility of child rising [18]. Scholars also suggested that women report poorer QOL because their illnesses may be taken less seriously and therefore they receive less social support than their male counterparts [31]. The result also showed females were less educated, more unemployed and had more opportunistic diseases like TB, depression, malnutrition and anemia than males which might lead to poor QOL. In this study social relationships health domain was not significantly different between men and women which was in line with the findings of other studies [15, 18, 22, 31]. Similarly, Spiritual domain was not significantly different between men and women though high quality of life than the other domains. The possible reasons for scoring high quality of life than the other domains could be that people tend to be spiritual and religious when confronted with problems that are beyond them; they engage in spiritual and religious reflections [32].

This study also indicated that illiterate individuals had significantly lower QOL in independence, social and environmental domains compared with literate counter parts. This was in concordance with a multi-country study among patients with HIV [6], and a study conducted in Ethiopia [23] stated that the less educated had lower QOL. Another study conducted in northern Ethiopia also reported illiterate People living with HIV/AIDS had lower physical and social domains of quality of life [22]. This might be due to literate individuals could have better financial resources, work capacity and access to quality health and social care.

This study also revealed that unemployed individuals had poor QOL in physical, psychological, social and environmental domains compared with employed individuals. This finding is consistent with other similar studies [13, 24, 25]. Study conducted in northern Ethiopia also reported that monthly income significantly associated with Physical, independence, social and environmental domains [22]. Similarly, PLWHA who received family support had significantly higher QOL in physical, psychological, social and spiritual domains as compared with who lacked family support. Studies also stated that lack of social support, lower level of education and income had associated with poor QOL [22, 33, 34]. This result may be attributable to the fact that psychosocial support could have increased personal satisfaction and positive entire effect in having good nutrition, self-care and better health [35].

Among females, residence was one of the significant predictor of their quality of life. Rural resident women showed lower QOL in independence and environmental domains than urban residents. A facility based cross sectional study from northern Ethiopia also found significant association between rural residence and poor quality of life [22]. This may be associated with the presence of relatively poor infrastructures, more financial constraints as well as high social stigma and discrimination in rural areas. In addition, females in rural areas engaged in more physical demanding work like farming as well their economic dependency on men compared to urban dwellers could limit access to better health services [21].

The study identified that physical, psychological, independence and environmental QOL domains were significantly reduced among undernourished HIV/AIDS patients. This finding was supported by the study from Nepal [17] and India [36]; that showed most of health related quality of life domains of PLWHA were significantly decreased as body mass index lowered. A study from South Africa also found that physical and independence health were affected by daily energy intake [10]. This was also strengthen by the outcome that better nutrition promote quality of life [37]. Physical health might be lowered because of under nutrition aggravates severity of HIV.AIDS symptoms. Poor psychological health could be due to fright that underweight may exposed their HIV status and get rejection from their family and social life or because of negative perception about their body image change due to underweight [38]. Poor independence health among malnourished HIV/AIDS patients might be due to decreased activity, mobility and work capacity.

In this study anemia co-morbidity in HIV patients was independent strong predictor for poor physical, psychological, independence and environmental health domains. This finding was consistent with a study conducted in India which found that lower hemoglobin level significantly affects five health quality of life domains except social domain [10]. Evidences also indicate that anemia among HIV/AIDS patients was correlated with high degree of disease advancement, diminished health related quality of life and high mortality [39–41]. Longitudinal study also discovered that increscent of hemoglobin levels through treatment will reduce blood transfusion necessities and enhance quality of life of people living with HIVAID [42].

The result also showed that tuberculosis co-infection was one of a strong determinant for decreased physical, psychological, independence, social, and environmental health among HIV/AIDS patients except their spiritual health. This find was supported by studies conducted in Ethiopia and in Nepal that stated Co-morbidity with tuberculosis in HIV infected patients deteriorates all six health related quality of life domains [11, 23]. Studies also described that HIV patients had poor QOL as compared to the general population [9] and lower QOL values were scored in tuberculosis patients [34, 43]. These findings indicate that the double burden from the two diseases have synergetic impact on overall health state of the patient.

The study identified that depressed PLWHA had significantly lower QOL in all domains as compared with HIV infected patients without depression in both genders. Studies also reported depression depreciates all health related quality of life categories and has been recognized as main determinant of alterations in overall health perceptions and psychological conditions in HIV infected people [12, 16, 17, 44] together with social care and psychotherapy practices [45, 46]. A study conducted in Ethiopia also identified that depression was associated with poor physical, social and environmental health [23]. In addition, lower quality of life scores were found in individuals with common mental disorder symptoms [26, 33, 47, 48]. Evidences revealed that QOL could be influenced by various determinants of psychological morbidity and depression is one of the most important of these factors [11, 49–51].

The study had limitations that should be acknowledged. This study was subject to selection bias since the respondents were ones who were actively seeking routine medical care selected from health facilities. They may not represent patients who do not come to the institution from the communities; therefore, generalizability is only for those who are on ART follow-up. Since interview administered questionnaires for HRQOL in the last 2 weeks were used interviewer and recall bias may be introduced to the study though pretested. The cross sectional nature of the study itself limits conclusions on QOL over a period of time and difficult to know cause- effect temporal relationships could not establish the circumstances resulting in low health related quality of life. Since there are many social factors that may alter people’s health related quality of life on HAART and might confound the findings of this study. we recommend further longitudinal studies triangulated with qualitative methods.

## Conclusions

The findings showed that females had significantly lower quality of life in physical, psychological, independence and environmental domains as compared with males, except social relationship and spiritual domains. The independent significant predictors of health related quality of life domains in both genders were education, occupation, malnutrition, depression, tuberculosis, anemia and family support. in addition, rural residence was also significantly associated with QOL domains in females. Therefore, to improve HRQOL of PLWHA due emphasis should be given to females and co-infected individuals improve socio-economic status and enhance integrated early detection and management of malnutrition, depression, tuberculosis and anemia among PLWHA in Ethiopia.

## Abbreviations

AIDS: Acquired immune deficiency syndrome
AOR: Adjusted odds ratio
ART: Antiretroviral therapy
CI: Confidence interval
HRQOL: Health related quality of life
PLWHA: People living with HIV/AIDS
QOL: Quality of life
TB: Tuberculosis
WHO: World Health Organization
